# C-Arylation reactions catalyzed by CuO-nanoparticles under ligand free conditions

**DOI:** 10.3762/bjoc.6.35

**Published:** 2010-04-15

**Authors:** Mazaahir Kidwai, Saurav Bhardwaj, Roona Poddar

**Affiliations:** 1 Green Research Laboratory, Department ofChemistry, University of Delhi, Delhi, India 110007

**Keywords:** active methylene compounds, C-arylation, heterogeneous catalyst, recyclability

## Abstract

CuO-nanoparticles were found to be an excellent heterogeneous catalyst for C-arylation of active methylene compounds using various aryl halides. The products were obtained in good to excellent yield. The catalyst can be recovered and reused for four cycles with almost no loss in activity.

## Introduction

Carbon-carbon (C–C) bond formation is one of the most important reactions in organic synthesis [1–3]. The resulting compounds formed from C–C coupling are valuable synthons in organic synthesis [4–7]. However, C-arylation reactions have not been investigated to the same extent as other C–C bond forming reactions.

A great deal of attention has been focused on the development of Pd catalyzed arylation [8–11]. Another important protocol involves arylation of activated methylene compounds mediated by copper salts [12–13]. Recently, proline has also been used along with CuI for C-arylation [14]. But some of the methods suffer from serious drawbacks and limitations, for example, long reaction times [15–16], the high cost of Pd catalysts [17–18] and the need for stoichiometric amounts of copper salts. In addition to these problems, the major drawback is that the majority of catalysts cannot be reused [19]. To overcome from these problems, we have investigated a new catalytic system for C-arylation.

Nanocrystalline metal oxides find numerous applications, e.g., as active adsorbents for gases [20], the destruction of hazardous materials [21] and the oxidation of volatile organic compounds [22]. In addition, metal oxide nanoparticles have been used as heterogeneous catalysts for various organic transformations [23–28]. The high reactivity of CuO-nanoparticles (CuO-np) is due to the high surface area of nanoparticles combined with unusual reactive morphologies. Moreover, heterogeneous catalysts are easy to separate and can be recycled. This is very beneficial for industrial process in the green chemistry domain.

In continuation with our research program to explore different methodologies for the synthesis of organic compounds [29–30] and the role of transition metal nanoparticles as catalysts in organic reactions [31–33], we now report the synthesis of 3-arylpentane-2,4-diones and diethyl 2-aryl-malonates using CuO-nanoparticles as a heterogeneous catalyst.

## Results and Discussion

**Scheme 1:**
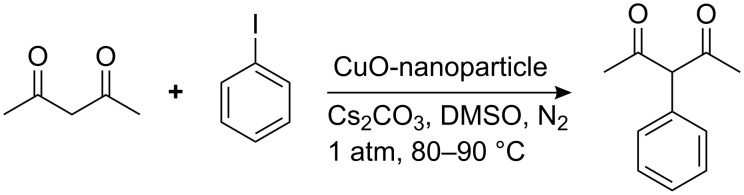
Synthesis of 3-phenylpentane-2,4-dione using CuO-nanoparticles.

The reaction was carried out several times in order to establish the optimum ratio of reactants. Iodobenzene and acetylacetone were employed as model substrates in a 1:3 ratio. When the iodobenzene (1 mmol) and acetylacetone (3 mmol) were stirred with Cs_2_CO_3_ (0.5 mmol) at 80 °C in DMSO, in the presence of CuO-nanoparticles (10 mol %), 3-phenyllpentane-2,4-dione was obtained in 80% yield (Scheme 1).

Other copper salts such as Cu(OAc)_2_ and ordinary CuO were found to be inferior to CuO-nanoparticles and gave low yields of 3-phenylpentane-2,4-dione. Moreover, Cu-nanoparticles which have been used for O-arylation and S-arylation [34–35], were not effective in the coupling reaction (Table 1).

**Table 1:** C-arylation reaction catalyzed by different Cu catalysts^a^.

Entry	Catalyst	Time (h)	Yield^b^ (%)

1	Cu(OAc)_2_	10	20
2	Cu-np	10	10
3	CuO	10	24
4	CuO-np	8	80

^a^Reaction conditions: acetylacetone (3 mmol), iodobenzene (1 mmol), 10 mol % of catalyst, Cs_2_CO_3_ (0.5 mmol), DMSO; temperature 80 °C; N_2_; 1 atm. ^b^Isolated and optimized yield.

**Figure 1:**
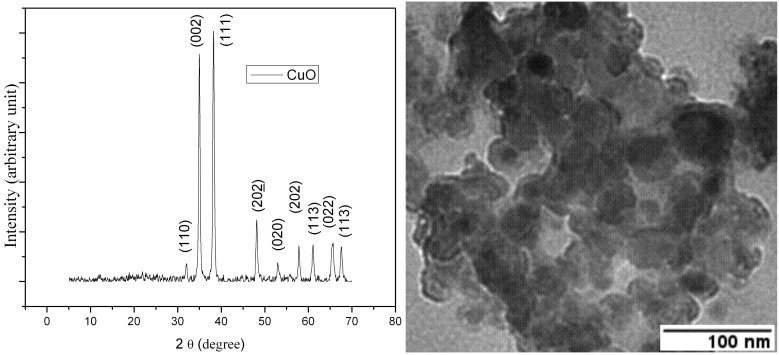
Powder X-ray diffraction pattern and TEM image of nano CuO (fresh).

In addition, catalytic activity of CuO-nanoparticles was evident since no product was formed in its absence. The increased catalytic activity of CuO-nanoparticles over the commercially available bulk CuO may be attributed to the higher surface area of CuO-nanoparticles. This is thought to be due to morphological differences which have been shown in the TEM image. The number of reactive sites on the surface is small in the case of larger crystallites and considerably greater in the case of smaller crystallites (Figure 1).

To investigate further the surface morphology of CuO-nanoparticles, powder XRD and TEM images were taken. Figure 1 shows the XRD pattern of CuO-nanoparticles in which diffraction peaks can be indexed to a monoclinic structure. The intense diffraction peak at an angle 38.4 shows index plane (111) which contains more basic sites with higher density as compared to bulk CuO.

During optimization of reaction conditions, the model reaction was carried out in different solvents. It was found that DMSO was the most effective solvent compared to the other solvents used as shown in Table 2. This is not surprising in view of the fact that the reaction intermediate is a carbanion and therefore will have a greater stability in a polar solvent.

**Table 2:** CuO-nanoparticles catalyzed coupling reaction of acetylacetone and iodobenzene in various solvents^a^.

Entry	Solvent	Time (h)	Yield^b^ (%)

1	DMSO	8	80
2	Toluene	15	8
3	THF	15	12
4	Acetonitrile	15	32

^a^Reaction conditions: acetylacetone (3 mmol), iodobenzene (1 mmol), 10 mol % CuO-nanoparticles, Cs_2_CO_3_ (0.5 mmol), solvent; temperature 80 °C; N_2_; 1 atm. ^b^Isolated and optimized yield.

Moreover, the advantage of the nanoparticles is that, unlike other catalysts, their use is not restricted by their solubility, as the nanoparticles can be dispersed in the desired solvent by agitation or slight sonication.

**Scheme 2:**
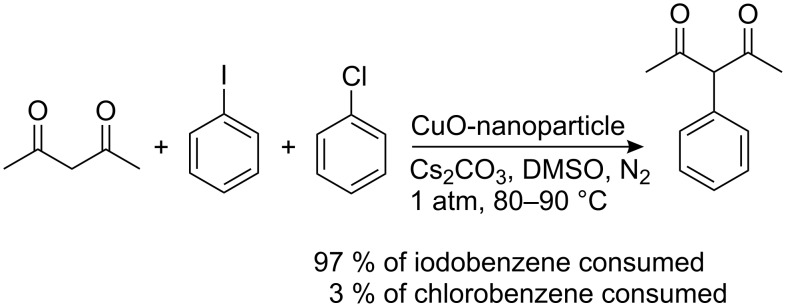
Synthesis of 3-phenylpentane-2,4-dione using CuO-nanoparticles.

When an equimolar mixture of chlorobenzene and iodobenzene was treated with acetylacetone under similar reaction conditions, the chlorobenzene was largely unreactive. This shows that the reaction is highly selective towards the halogen present in the aryl halide (Scheme 2).

To study the scope of this procedure, acetylacetone was reacted with various aryl halides and gave the corresponding products in 78–83% yield. It was observed that aryl halides having electron withdrawing groups showed greater reactivity and gave good yield of products compared to aryl halides having electron donating groups. All these results are summarized in Table 3.

**Table 3:** C-arylation of acetylacetone using different aryl halides^a^

Entry	Aryl halide	Product	Time (h)	Yield^b^ (%)

1	iodobenzene	3-phenylpentane-2,4-dione	8	80
2	bromobenzene	3-phenylpentane-2,4-dione	10	79
3	*p*-nitroiodobenzene	3-(4-nitrophenyl)-pentane-2,4-dione	6	83
4	*p*-methyliodobenzene	3-*p*-tolylpentane-2,4-dione	10	78
5	*m*-trifluoromethyliodobenzene	3-(3-trifluromethyl-phenyl)-pentane-2,4-dione	7	81
6	1-iodo-2-methylbenzene	3-*o*-tolyl-pentane-2,4-dione	11	76
7	1-iodo-4-methoxybenzene	3-(4-methoxy-phenyl)-pentane-2,4-dione	12	75

^a^Reaction conditions: acetylacetone (3 mmol), aryl halide (1 mmol), 10 mol % CuO-nanoparticles, Cs_2_CO_3_ (0.5 mmol), DMSO; temperature 80 °C; N_2_; 1 atm. ^b^Isolated and optimized yields.

**Scheme 3:**
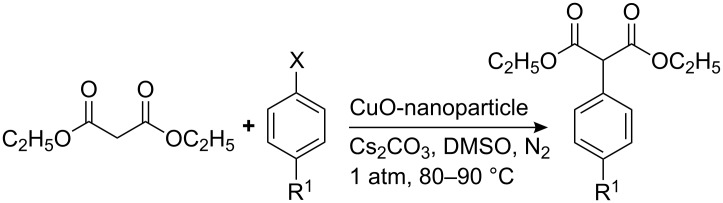
Synthesis of diethyl 2-aryl-malonate using CuO-nanoparticles.

The above results encouraged us to investigate further reactions. Under similar reaction conditions, diethyl malonate was treated with iodobenzene to give the desired product in 78% yield (Scheme 3). The reaction was then repeated with a variety of aryl halides. The results are summarized in Table 4.

**Table 4:** C-arylation of diethyl malonate using different aryl halides^a^.

Entry	Aryl halide	Product	Time (h)	Yield^b^ (%)

1	iodobenzene	2-phenylmalonic acid diethylester	9	78
2	bromobenzene	2-phenylmalonic acid diethylester	11	76
3	*p*-nitroiodobenzene	2-(4-nitrophenyl)-malonic acid diethylester	6	81
4	*p*-methyliodobenzene	2-*p*-tolylmalonic acid diethylester	12	76
5	*m*-trifluoromethyliodobenzene	2-(3-trifluromethyl-phenyl)-malonic acid diethylester	7	80
6	1-iodo-2-methylbenzene	2-*o*-tolylmalonic acid diethylester	13	75
7	1-iodo-4-methoxybenzene	3-(4-methoxy-phenyl)-malonic acid diethylether	14	74

^a^Reaction conditions: diethyl malonate (3 mmol), aryl halide (1 mmol), 10 mol % CuO-nanoparticles, Cs_2_CO_3_ (0.5 mmol), DMSO; temperature 80 °C; N_2_; 1 atm. ^b^Isolated and optimized yields.

**Figure 2:**
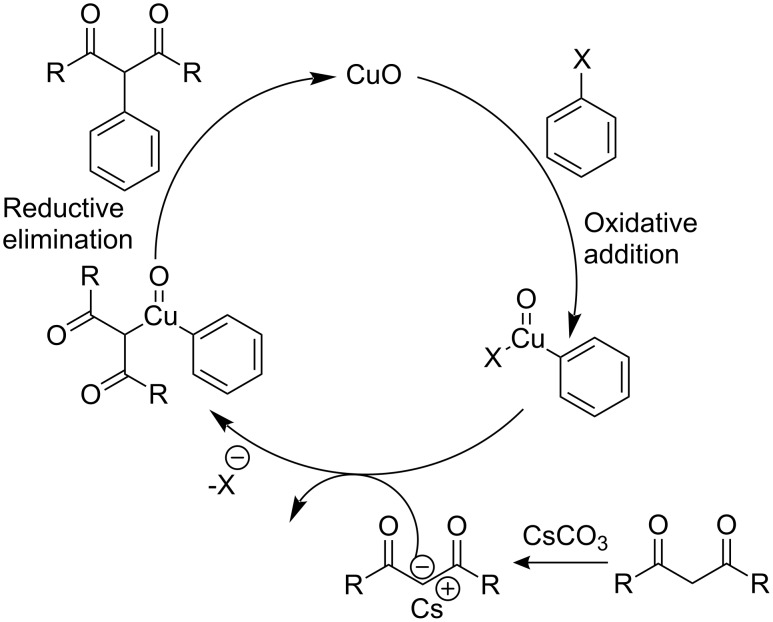
Proposed reaction pathway for the CuO-nanoparticles catalyzed C–C coupling reaction.

The observed decrease in reactivity in the order *p*-nitroiodobenzene > iodobenzene > *p*-methyliodobenzene > 1-iodo-4-methoxybenzene suggests that the reaction proceeds by oxidative addition followed by reductive elimination. In addition to this, the order of reactivity suggests that aryl halides having electron withdrawing groups stabilize the transition state which corresponds to good yields with a short reaction time in comparison to aryl halides having electron donating groups (Figure 2).

**Figure 3:**
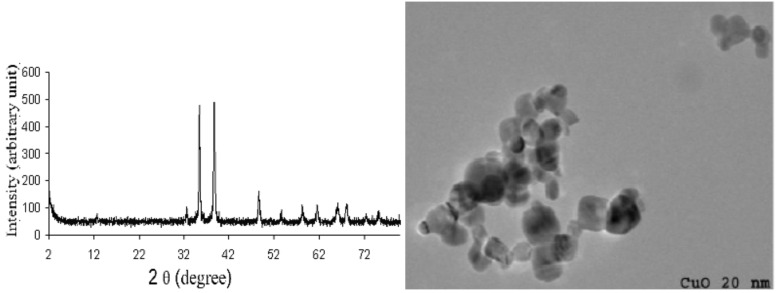
Powder X-ray diffraction pattern and TEM image of recycled CuO-nanoparticles.

The reaction was then carried out with recycled catalyst. The CuO-nanoparticles were recovered by centrifugation of the reaction mixture and washed thoroughly with ethyl acetate. The resulting nanoparticles could be reused for several cycles without any significant loss of activity. In our study, we used same nanoparticles three times and the results are summarized below (Table 5). The TEM and XRD analysis of recycled CuO-nanoparticles, after the fourth run showed that particles are identical in shape and size, hence CuO-nanoparticles are unchanged during the reaction (Figure 3).

**Table 5:** Recycle studies of nano-CuO for C-arylation reaction^a^.

Run	Time (h)	Yield^b^ (%)

1	8	80
2	8	78
3	8	77
4	8	76

^a^Reaction conditions: acetylacetone (3 mmol), iodobenzene (1 mmol), 10 mol % CuO-nanoparticles, Cs_2_CO_3_ (0.5 mmol), DMSO; temperature 80 °C; N_2_; 1atm. ^b^Isolated and optimized yields.

## Conclusion

An efficient, facile and economical method for synthesis of 3-arylpentane-2,4-diones (Table 3) and diethyl 2-aryl-malonates (Table 4) has been developed using CuO-nanoparticles as the catalyst. The products were obtained in moderate to good yields and the catalyst can be recycled up to four cycles with almost consistent activity. The present protocol represents a simple and remarkably active catalytic system to catalyze Ullmann-type C–C bond formation, which potentially offers an efficient protocol for accessing a variety of α-arylated dicarbonyl compounds.

## Experimental

### General

The materials were purchased from Sigma-Aldrich and Merck and were used without further purification. Mass spectra were recorded in a TOF-mass spectrometer model no. KC455. ^1^H NMR and ^13^C NMR spectra were recorded on a Bruker spectrospin at 300 MHz and 75 MHz, respectively. All ^1^H NMR and ^13^C NMR spectra were run in CDCl_3_ and chemical shifts are expressed as ppm relative to internal Me_4_Si. Elemental analyses were performed using Heraeus CHN-Rapid Analyzer. Powder X-ray diffraction measurements were carried out on a Bruker D8 Discover HR-XRD instrument using Cu Kα radiation (λ = 1.54184 Å).

### Experimental Section

CuO-nanoparticles (10 mol %) were added to the mixture of iodobenzene (1 mmol), acetylactone (3 mmol) and (0.5 mmol) of Cs_2_CO_3_ in DMSO (2 ml) under nitrogen atmosphere. The resulting reaction mixture was stirred at 80 °C for 8 h. Progress of the reaction was continuously monitored by TLC. After completion of reaction, the catalyst was recovered by centrifugation. The nanoparticles, were first washed with distilled methanol to remove some of the Cs_2_CO_3_, then several times with ethyl acetate and dried in an oven overnight at 150 °C. The filtrate was poured into 1N HCl and extracted with EtOAc. The combined organic layers were washed with brine, dried over anhydrous Na_2_SO_4_ and concentrated at reduced pressure. The residue was chromatographed to afford pure 3-phenyl-2,4-pentadione (Entry 1, Table 3). From the spectral data it was observed that some products show 1,3-keto-enol tautomerism. The structures of all the products were unambiguously established on the basis of their spectral properties (^1^H NMR, MS and ^13^C NMR).

## Supporting Information

File 1 Typical procedure and characterization data of prepared compounds
