# Supplementary material for: C-Arylation reactions catalyzed by CuO-nanoparticles under ligand free conditions
Source: Beilstein J Org Chem. 2010 Apr 15;6:35. doi: 10.3762/bjoc.6.35 (PMC2874413; doi:10.3762/bjoc.6.35)
Supplement: File 1 — Typical procedure and characterization data of prepared compounds [file Beilstein_J_Org_Chem-06-35-s001.pdf]

Supporting information for

C-Arylation reactions catalyzed by CuO-nanoparticles under ligand free conditions

Mazaahir Kidwai*, Saurav Bhardwaj and Roona Poddar

Green Research Laboratory, Department of Chemistry, University of Delhi, Delhi, India 110007

Email: Mazaahir Kidwai - kidwai.chemistry@gmail.com

* Corresponding author

Experimental

General

The materials, procured from Sigma-Aldrich and Merck, were used without further purification. Mass spectra were recorded in a TOF-mass spectrometer model no. KC455. ^1H NMR and ^{13}C NMR spectra were recorded on a Bruker spectrospin at 300 MHz and 75 MHz, respectively. All NMR spectra were run in $CDCl_3$ and chemical shifts are expressed as ppm relative to internal Me_4Si. Powder X-ray diffraction measurements were performed on a Bruker D8 Discover HR-XRD instrument using Cu K_α radiation (λ = 1.54184 Å).

Typical Procedure

CuO-nanopaticles were added to a mixture of iodobenzene (1m.mol), acetylacetone (3m.mol) and (0.5 mmol) Cs_2CO_3 in DMSO (2 ml). The resulting reaction mixture was stirred at 80 °C for 9h. Progress of the reaction was continuously monitored by TLC. After the completion of reaction, the catalyst was recovered by centrifuge and washed several times with ethyl acetate. The filtrate was poured into 1N HCl and extracted with EtOAc. The combined organic layers were washed with brine, dried over anhydrous Na_2SO_4 and concentrated. The residue was chromatographed to afford the pure 3-arylpentane-2,4-dione. From the spectral data it was found that some products show 1,3- keto-enol tautomerism. The structures of all the products were unambiguously established on the basis of their spectral properties (^1H NMR and ^{13}C NMR).

3-Phenylpentane-2,4-dione (Table 3, entry 1)
Yellowish oil. ^1H NMR (300 MHz, TMS, $CDCl_3$): δ 1.98 (s, 3H, CH_3), 2.11 (s, 3H, CH_3), 6.77–7.86 (m, 5H, φ), 12.50 (s, 1H,OH). ^{13}C NMR (75 MHz, $CDCl_3$): δ 19.60, 29.25, 116.53, 126.96, 128.70, 134.47, 163.31, 200.48. *m/z* (GC-MS): 176.06 (M^+). Elemental analysis; Calculated C, 74.98; H, 6.86; O, 18.16. Observed C, 74.80; H, 6.96; O, 18.24.

3-Phenylpentane-2,4-dione (Table 3, entry 2)
Yellowish oil. ^1H NMR (300 MHz, $CDCl_3$): δ 1.98 (s, 3H, CH_3), 2.20 (s, 3H, CH_3), 6.86–7.86 (m, 5H, φ), 12.50 (s, 1H,OH). ^{13}C NMR (75 MHz, TMS, $CDCl_3$): δ 19.60, 29.25, 116.53, 126.96,

128.70, 134.47, 163.31, 200.48. *m/z* (GC-MS): 176.08 (M^+). Elemental analysis; Calculated C, 74.98; H, 6.86; O, 18.16. Observed C, 74.80; H, 6.96; O, 18.24.

3-(4-Nitrophenyl)-pentane-2,4-dione (Table 3, entry 3)
Yellow solid. ^{1}H NMR (300 MHz, $CDCl_3$): δ 2.12 (s, 6H, CH_3), 4.12 (s, 1H, C_3-H), 7.14 (d, J = 8.4, 2H,φ), 8.23 (d, J = 8.4, 2H, φ). *m/z* (GC-MS): 220.96 (M^+). Elemental analysis Calculated C, 59.73; H, 5.01; N, 6.36; O, 28.93 Observed C, 60.04; H, 5.09; N, 6.36; O, 28.93.

3-*p*-Tolyl-pentane-2,4-dione (Table 3, entry 4)
Yellowish oil. ^{1}H NMR (300 MHz, $CDCl_3$): δ 1.94 (s, 3H, CH_3), 2.10 (s, 3H, CH_3), 7.92 (d, 2H, J = 8.1, φ), 8.11 (d, J = 8.1, 2H, φ), 12.74 (s, 1H, OH). *m/z* (GC-MS): 190.10 (M^+). Elemental analysis Calculated C, 75.86; H, 7.32; O, 16.82 Observed C, 74.86; H, 7.24; O, 16.80.

3-(3-Trifluromethylphenyl)-pentane-2,4-dione (Table 3, entry 5)
Yellowish oil. ^{1}H NMR (300 MHz, $CDCl_3$): δ 2.10 (s, 6H, CH_3), 3.85 (s, 1H), 7.32–7.53 (m, 3H, φ), 7.84 (s, 1H, φ). ^{13}C NMR (75 MHz, TMS, $CDCl_3$): δ 20.36, 21.27, 116.42, 119.15, 123.75, 124.29, 128.31, 129.31, 130.99, 134.93, 166.45, 202.08. *m/z* (GC-MS): 244.20 (M^+). Elemental analysis Calculated C, 59.02; H, 4.54; O, 13.10. Observed C, 60.01; H, 3.50; O, 13.13.

3-*o*-Tolyl-pentane-2,4-dione (Table 3, entry 6)
Yellowish oil. ^{1}H NMR (300 MHz, TMS, $CDCl_3$): δ 1.97 (s, 3H, CH_3), 2.12 (s, 3H, CH_3), 6.77–7.86 (m, 5H, φ), 12.50 (s, 1H,OH). (GC-MS): 190.10 (M^+). Elemental analysis Calculated C, 75.86; H, 7.32; O, 16.82 Observed C, 74.86; H, 7.24; O, 16.80.

3-(4-Methoxyphenyl)-pentane-2,4-dione (Table 3, entry 7)
Yellowish oil. ^{1}H NMR (300 MHz, TMS, $CDCl_3$): δ 2.12 (s, 3H, CH_3), 3.72 (s, 3H), 3.82 (s, 1H), 6.78 (d, 2H, J = 8.1, φ), 7.86 (d, 2H, J = 8.1, φ). (GC-MS): 206.03 (M^+). Elemental analysis Calculated 69.88; H, 6.84; O, 23.27 Observed C, 68.86; H, 6.94; O, 23.20.

2-Phenylmalonic acid diethylester (Table 4, entry 1)
Light-yellow oil. ^{1}H NMR (300 MHz, $CDCl_3$): δ 1.30 (t, J = 7.3, 6H, CH_3), 4.32 (q, J = 7.3, 4H, CH_2), 4.62 (s, 1H), 7.21–7.80 (m, 5H, φ). ^{13}C NMR (75 MHz, TMS, $CDCl_3$): δ 13.62, 53.12, 59.54, 124.89, 125.94, 128.80, 134.93, 170.90. *m/z* (GC-MS): 236.10 (M^+). Elemental analysis Calculated C, 66.09; H, 6.83; O, 27.09. Observed C, 65.97; H, 6.24; O, 26.80.

2-Phenylmalonic acid diethylester (Table 4, entry 2)
Light-yellow oil. ^{1}H NMR (300 MHz, $CDCl_3$): δ 1.30 (t, J = 7.3, 6H, CH_3), 4.32 (q, J = 7.3, 4H, CH_2), 4.60 (s, 1H), 7.22–7.70 (m, 5H, φ). ^{13}C NMR (75 MHz, TMS, $CDCl_3$): δ 13.62, 53.12, 59.54, 124.89, 125.94, 128.80, 134.93, 170.90. *m/z* (GC-MS): 236.10 (M^+). Elemental analysis Calculated C, 66.09; H, 6.83; O, 27.09. Observed C, 65.97; H, 6.24; O, 26.89.

2-(4-Nitrophenyl)-malonic acid diethylester (Table 4, entry 3)
Dark-yellow solid. ^{1}H NMR (300 MHz, $CDCl_3$): δ 1.30 (t, J = 7.3, 6H, CH_3), 4.32 (q, J = 7.3, 4H, CH_2), 4.92 (s, 1H),7.25 (d, J = 8.2, 2H,φ), 7.98 (d, J = 8.2, 2H,φ). *m/z* (GC-MS): 281.20 (M^+). Elemental analysis, Calculated C, 55.51 ; H, 5.38; N, 4.98; O, 34.13. Observed C, 55.50; H, 5.36; N, 4.90; O, 33.10.

2-*p*-Tolyl-malonic acid diethylester (Table 4, entry 4)

Yellowish oil. ^{1}H NMR (300 MHz, $CDCl_3$): δ 1.30 (t, J = 7.3, 6H, CH_3), 2.35 (s, 3H, φ-CH_3), 4.21 (q, J = 7.3, 4H, CH_2), 4.54 (s, 1H), 7.10 (d, J = 8.3, 2H,φ), 7.72 (d, J = 8.3, 2H, φ). *m/z* (GC-MS): 250.20 (M^+). Elemental analysis, Calculated C, 67.18; H, 7.26; O, 25.57. Observed C, 66.84; H, 7.45; O, 25.20.

2-(3-Trifluromethylphenyl)-malonic acid diethylester (Table 4, entry 5)

Light-yellowish oil. ^{1}H NMR (300 MHz, $CDCl_3$): δ 1.30 (t, J = 7.3, 6H, CH_3), 4.21 (q, J = 7.3, 4H, CH_2), 4.67 (s, 1H), 7.21–7.50 (m, 3H, φ), 7.73 (s, 1H, φ). ^{13}C NMR (300 MHz, TMS, $CDCl_3$): δ 13.83, 53.36, 60.91, 118.49, 124.89, 125.92, 128.80, 130.87, 170.90. *m/z* (GC-MS): 304.05 (M^+). Elemental analysis Calculated C, 55.26; H, 4.97; O, 18.43. Observed C, 55.20; H, 75.08; O, 18.40.

2-*o*-Tolyl-malonic acid diethylester (Table 4, entry 6)

Yellowish oil. ^{1}H NMR (300 MHz, $CDCl_3$): δ 1.30 (t, J = 7.3, 6H, CH_3), 2.36 (s, 3H, CH_3), 4.21 (q, J = 7.3, 4H, CH_2), 4.52 (s, 1H),7.10–7.72 (m, 4H, φ). *m/z* (GC-MS): 250.20 (M^+). Elemental analysis, Calculated C, 67.18; H, 7.26; O, 25.57. Observed C, 60.84; H, 7.40; O, 25.40.

3-(4-Methoxyphenyl)-malonic acid diethylester (Table 4, entry 7)

Colourless oil. 1H NMR (300 MHz, $CDCl_3$): δ, 1.26 (t, J = 7.0 Hz, 6H), 3.80 (s, 3H), 4.56 (s, 1H), 4.24–4.17 (m, 4H), 6.89 (d, J = 8.5 Hz, 2H), 7.32 (d, J = 8.0 Hz, 2H). *m/z* (GC-MS): 266.1 (M^+).
